# Concurrent Hand and Penile Gangrene following Prolonged Warfarin Use; a Case Report

**Published:** 2017-06-21

**Authors:** Fatemeh Mahdizadeh, Saeed Safari

**Affiliations:** 1 Department of Emergency Medicine, Ilam University of Medical Sciences, Ilam, Iran.; 2 Department of Emergency Medicine, Shahid Beheshti University of Medical Sciences, Tehran, Iran.

**Keywords:** Warfarin, penile diseases, gangrene, anticoagulants, necrosis, extremities

## Abstract

Warfarin induced skin necrosis (WISN) is a rare but important side effect of warfarin. Early diagnosis may lessen the amount of permanent tissue damage and can prevent progression to full thickness skin necrosis. So, physicians should be aware of such a complication. Screening for protein C or S or anti-thrombin deficiencies, or presence of anti-phospholipid antibodies before beginning warfarin therapy, could be helpful to avoid high levels of international normalized ratio (INR). Here, we report a 54-year-old man who presented to the emergency department with acral and penile gangrene following prolonged use of warfarin.

## Introduction

Warfarin is widely used as an anticoagulant agent. Warfarin induced skin necrosis (WISN) is an uncommon but serious condition, occurring in 1:10000 patients who receive this drug, with a female/male ratio of 1:4 (1). Skin necrosis has been reported in 0.17% of patients affected with side effects of warfarin (2, 3). The majority of cases present in the first month of treatment, but late onset WISN may also occur up to 15 years after starting the treatment (4, 5). In this report we describe a patient who had developed late onset warfarin-induced skin necrosis, concurrently in acral and penile area.

## Case presentation

**Figure 1:**
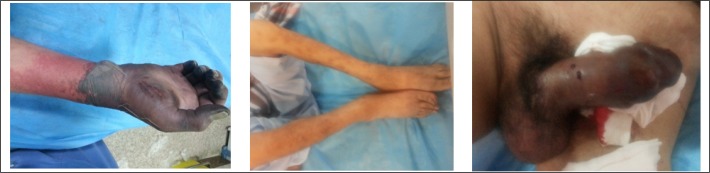
Patient’s lesions in the right upper and lower extremities as well as penis.

The patient was a 54-years-old male presented with penis and right upper extremity pain, black discoloration and swelling for the past two days. The patient was a known and treated case of deep vein thrombosis from 6 years before and he was taking warfarin intermittently during this time. He was opium addicted. The patient was a cachectic man. He was completely alert (GCS =15). On arrival to the emergency department, pulse rate was 90 per minute, respiratory rate was 18 per minute, blood pressure was 115/70 mmHg and body temperature was 37.8 °C. Distal part (about one third) of his right forearm was swollen, tender and black discolored and a bulla around the wrist was seen. Right lower extremity was edematous. Distal pulses of lower limbs were palpable but diminished because of edema. Also the penis was extremely tender, swollen, erected and dark discolored. Figure 1 shows the patient’s lesions. His primary lab results were as follows:

White Blood Cell=6500 cells/µL, Hemoglobin=6.8 gr/dL, Hematocrit =20.3, Platelet=269000 cells/µL, Prothrombin Time (PT) =28 s, Partial thromboplastin time (PTT)=43 s, international normalized ratio (INR)=4.6, Blood Urea Nitrogen=10 mg/dl, Creatinine=0.7 mg/dl, Calcium=8 mg/dl, Phosphorous=2.7 mg/dl, Total Protein=3.9 mg/dl, Albumin=1.8 mg/dl, Aspartate Aminotransferase (AST)=16 U/L, Alanine Aminotransferase (ALT)=13 U/L, Lactate Dehydrogenase (LDH)=429 U/L, Creatine Phosphokinase (CPK)=83 U/L. HBs-Ag, HCV-Ab and HIV-Ab were all reported as negative.

Ultrasonography of right hand revealed subcutaneous edema and fluid collection. A hematoma was reported in dorsal area at the level of first metacarpophalangeal joint. Arterial and venous duplex of right upper extremity were normal. Also CT angiography of aorta to distal of right upper limb was normal without cut off. Ultrasonography of testicles revealed right extra-testicular hematoma, left atrophic testis and edema of scrotum. Duplex of both testicles were normal. Warfarin was discontinued and correction of coagulation tests was done by replacement of fresh frozen plasma and vitamin K. Also cefazolin was ordered intravenously every 8 hours and 16-French foley catheter was fixed. Partial penectomy and urethroplasty of anterior duct was done approximately two months after admission and then amputation of right hand above the wrist was performed. Histological findings of portion of penile shaft confirmed subtotal necrosis and areas of old and fresh hemorrhage and hematoma. The patient was stable and discharged four days after surgery. Surgeons advised him to follow up in clinic.

In our patient neither anti-phospholipid antibodies nor low levels of protein C or S were measured and nor heparin induced thrombocytopenia (HIT) was considered.

## Discussion

WISN begins as localized paresthesia with an erythematous flush, progresses to petechial and hemorrhagic bulla and may eventually result in full thickness necrosis (6). It usually involves areas with more subcutaneous fat content such as breasts, thighs, and buttocks (5, 7).

Although the pathogenesis of the disease is still unknown, there were numerous reports of inherited or functional deficiency of protein C, protein S or Factor V Leiden, antithrombin III, hyperhomocysteinemia or in association with HIT and anti-phospholipid syndrome. Another theory for explaining the pathophysiology of WISN is hypersensitivity reaction or a direct toxic effect (1, 2, 8). Obesity, premenopausal age, viral infections, hepatic disease, and drug interactions are mentioned as predisposing factors (1). 

The lesions must be differentiated from necrotizing fasciitis, venous gangrene, decubitus ulcer, and hematoma that are more common (1, 2).

WISN is usually diagnosed clinically, based on patient’s symptoms, lesion appearance and history of recent warfarin therapy (4). Determining protein C and S levels for assessing predisposing factors and skin biopsy can aid in diagnosis. Histology typically shows diffuse microthrombosis within dermal and subcutaneous capillaries, venules and deep veins, with endothelial cell damage resulting in ischemic skin necrosis and red blood cell extravasation (9).

The lack of perivascular inflammation and arteriolar thrombosis, differentiates WISN from the vasculitis (4, 9).

Treatment includes discontinuing warfarin, although it has not been shown to alter outcome, starting heparin, administrating vitamin K, fresh frozen plasma (FFP) or pure activated protein C because of its low level. Long-term treatment includes local wound care and observation of the wound until healing. Skin grafting, surgical debridement and amputation may be necessary in severe limb gangrene (4, 8). 

WISN should be suspected in all patients who undergo over-warfarinization, even with an initially normal coagulation profile. Prompt diagnosis and discontinuation of warfarin are crucial for the prognosis (1, 3). Necrosis may be prevented by identifying high risk patients and avoiding high dose of warfarin in high risk patients (1, 10). Several recommendations for preventing WISN have been advanced: 1) Heparin should be continued until the INR is near the therapeutic range as a result of the warfarin therapy and vitamin K dependent clotting factors have been consumed; 2) Standard or low dose warfarin should be used instead of initial large loading doses; 3) A clinician should be cautious when advancing the dosage of warfarin (6).

## Conclusion

WISN, while rare, is an important complication following warfarin administration. Therefore, physicians should be aware. Screening for protein C or S or anti-thrombin deficiencies, or presence of anti-phospholipid antibodies before beginning warfarin therapy, avoiding high INR level and finally, early diagnosis may lessen the amount of permanent tissue damage and can prevent progression to full thickness skin necrosis.
